# Ophthalmology Academic Leagues: making an impact on Medical Education

**DOI:** 10.5935/0004-2749.20200108

**Published:** 2024-02-11

**Authors:** Giovana Rosa Gameiro, Gustavo Rosa Gameiro

**Affiliations:** 1 Medical School, Universidade Estadual de Londrina, Londrina, PR, Brazil. Former president (2019) of the Brazilian Association of Ophthalmology Academic Leagues (ABLAO); 2 Department of Ophthalmology and Visual Sciences, Escola Paulista de Medicina, Universidade Federal de São Paulo, São Paulo, SP, Brazil. Former president (2016-2017) of the Brazilian Association of Ophthalmology Academic Leagues (ABLAO); 3 Health Education, Faculty Development Center (CEDEM), Faculdade de Medicina FMUSP, Universidade de São Paulo, São Paulo, SP, Brazil

With a rich history dating back to 1920, Medical Academic Leagues (MAL) in Brazil play an important and innovative role in medical education, research activities, and extension projects^(1)^. Medical Academic Leagues consist of students organizations with the aim to promote in-depth learning of a certain field and health advocacy, as well as to stimulate the development of valuable skills for future physicians, such as leadership, communication, teamwork abilities, critical thinking, and proactivity^(1^,^2)^.

Recently, with an increase in both the number and impact of MAL in Brazil, national associations were created in order to foster the integration and exchange of ideas between these academic organizations across the country. In this scenario, the Brazilian Association of Medical Academic Leagues (ABLAM) was created in 2005, vinculated to the Brazilian Medical Association (AMB), and today includes more than 4000 affiliated leagues^(2)^. After the establishment of ABLAM, national associations of Academic Leagues of determined medical specialties, such as Ophthalmology, General Surgery, Cardiology and Family Medicine were then created or strengthened.

The Brazilian Association of Ophthalmology Academic Leagues (ABLAO) was founded in 2013, during the 1^st^ National Meeting of Ophthalmology Academic Leagues, which occurred during the XVIII International Congress of the Brazilian Society of Ophthalmology^(3)^. The impact of ABLAO has grown consistently in recent years, and nowadays, over 100 Ophthalmology Leagues from all regions of Brazil are represented by this organization. With a mission of propagating knowledge and promoting the political, scientific, and social integration of Brazilian medical students interested in Ophthalmology^(4)^, ABLAO has conquered a relevant space in important Ophthalmology meetings in Brazil, including the following: the Brazilian Congress of Ophthalmology (CBO), Ophthalmology Congress of the University of São Paulo (COUSP), Brazilian Congress of the Brazilian Society of Contact Lenses, Cornea and Refractometry (SOBLEC), International Congress of Cataract and Refractive Surgery (BRASCRS), International Congress of the Brazilian Society of Ophthalmology (SBO), and the Moacyr Álvaro International Symposium (SIMASP). The inclusion of a session designed by students for students in these conferences increases the visibility of ABLAO, and encourages more students to participate in these events and in Academic Leagues.

The first Ophthalmology League in Brazil was created in 1977 at UNICAMP, as an initiative to complement the Ophthalmology curriculum of the university^(5)^. Currently, most Ophthalmology Leagues have extracurricular activities that involve theoretical classes, practical activities in the outpatient clinic or emergency room, participation in community projects, and research initiatives^(4^,^5)^. Essentially, the main goal is to empower students to become more prepared and well-trained doctors in the future, regardless of their specialty, as well as to bring them closer to their communities so they are reminded of the crucial role they play in the society^(1^,^4^,^5)^.

A multicentric research study conducted by ABLAO’s former board of directors in 2019 confirmed the positive impacts of Ophthalmology Academic Leagues on medical education. In this study, with a sample of 242 random medical students from over 12 Brazilian states, Ferreira et al. (2019) found that only 31% of the students felt confident about treating and/or referring to a specialist a patient who presents with an ocular complaint. Moreover, only 43.4% of the students were reported to have had the option of undertaking an elective clerkship in Ophthalmology in their medical schools, while 93% of the students had the opportunity to join an Ophthalmology Academic League^(4)^. These results show that future general practitioners tend to have an insufficient ophthalmic knowledge, and that a considerable number of medical institutions have a lack of practical opportunities in Ophthalmology to offer. Therefore, the presence of Ophthalmology Academic Leagues could be beneficial to the students, who could gain more exposure to the specialty, and acquire more solid training and practical skills in the field.

Another positive outcome of the participation in Ophthalmology Leagues during medical school is that it enables students to get to know the specialty and to take part in activities throughout their medical course, instead of having to wait for the subject to be taught in the later years of medical school. In this way, proactive students can get a broader exposure to the field, connect with residents and attendings and have more real experiences before choosing their future career path in medicine. In a recent publication regarding the motivating factors behind the choice of Ophthalmology as a career among Brazilians, 77.7% of the participants in the survey declared that they chose Ophthalmology after being exposed to the area in medical school^(6)^. However, even though taking part in Ophthalmology Leagues can certainly influence students to pursue such specialty, it does not necessarily mean that the participation in these academic organizations is restricted to students who are interested in applying for Ophthalmology residencies in the future. The previously cited multicentric study supports this idea: while 43% of the students who participated in the survey were involved in an Ophthalmology Academic League, only 22.3% of the total sample intended to pursue a career in Ophthalmology^(4)^.

Ultimately, Medical Academic Leagues are based on teaching, research, and outreach. These independent student associations reflect modern changes in the teaching-learning scenarios, with the implementation of new active methodologies in the educational system. In this way, students have become protagonists of their own learning, which bolsters the importance of selfmotivation to pursue a better education and create new learning opportunities with extracurricular activities^(7)^. Integrating innovative teaching activities with the social needs of the community contributes to the interdisciplinary education of highly skilled physicians, as well as foments research and health promotion initiatives^(1^,^2)^. These are the principles that ABLAO has been nurturing since its creation, and hopefully will continue to do so for many years to come.

## References

[1] Matheus BT. (2019). The important role of academic leagues (extensions) in Brazilian
medical education. Rev Assoc Med Bras.

[2] de Bastos ML, Trajman A, Teixeira EG, Selig L, Teixeira Belo MT. (2012). O papel das ligas acadêmicas na formação
profissional. J Bras Pneumol.

[3] Kato JM, Albuquerque GP, Yeh CO, Resende MF, Barros MF, Portes AL (2015). Associação Brasileira de Ligas Acadêmicas de
Oftalmologia: perspectivas e desafios. Rev Med.

[4] Ferreira MA (2019). Perfil multicêntrico do acadêmico de medicina e
suas perspectivas sobre o ensino da oftalmologia. Rev Bras Oftalmol.

[5] Kara José AC, Passos LB, Kara José FC, Kara José N. (2007). Ensino extracurricular em Oftalmologia: grupos de estudos / ligas
de alunos de graduação. Rev Bras Educ Med.

[6] Gameiro GR, Darcie ALF, Hazaki D, Gameiro GR, Carricondo PC. (2019). Why ophthalmology? Analysis of the motivating factors influencing
the choice of ophthalmology as a career among different generations in
Brazil. Clinics (Sao Paulo).

[7] Gameiro GR, Silva HB. (2018). Breaking paradigms: students in perspective. Clinics (Sao Paulo).

